# Innovation and the publishing gambit

**DOI:** 10.1308/rcsann.2024.0102

**Published:** 2024-11-01

**Authors:** B Rogers

**Figure:**
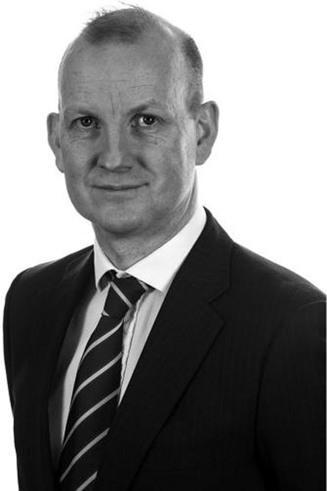
**B Rogers**, Editor-in-Chief *Annals* of the Royal College of Surgeons of England

The last issue of the *Annals* in 2024 focuses on innovation to complement the recent Future Surgery conference, supported by the Royal College of Surgeons of England.^1^ Four initial papers provide an excellent overview of robotic surgery in colorectal and gastrointestinal surgery. Thrikandiyur *et al* present a systematic review, meta-analysis and meta-regression of randomised trials considering the postoperative outcomes in patients undergoing robotic or laparoscopic surgery.^2^ The results indicate that robotic surgery is associated with an increasing safety profile for patients. May-Miller *et al* conducted a survey of the uptake of upper gastrointestinal robotic surgery in the UK, and in particular tertiary cancer resection centres, with conclusions showing that robotic gastrointestinal surgery is likely to become the predominant surgical approach in the future, with both patient and surgeon benefits.^3^ Further papers consider the use of artificial intelligence in paediatric fracture diagnosis and the performance of large language models in the MRCS examination! As always, the innovation edition provides readers with a wide and varied choice.

In line with the theme of innovation, we come to the end of the first year of the *Annals* as a gold open access journal.^4^ There were concerns and doubts around this change, which I fully understand. However, the scientific publishing landscape is transforming rapidly, and this move has enabled the *Annals* to maintain and enhance its reputation.

The article processing charge waiver for articles whose first or senior authors are fellows, members or affiliates of the Royal College of Surgeons of England provides a clear benefit to the wider membership. In metrics, full-page reads of *Annals* articles are approaching one million for the calendar year, with submissions maintained at over 500 per annum and an overall article acceptance rate of 21%. These figures compare favourably with larger commercially-funded specialty journals. The *Annals* continues to hold a unique position in medical publishing: a pan-surgical journal affording the opportunity to attract and publish research on topics that would be of limited interest to the readership of more specialised publications.

A prime example of the ability of the *Annals* to publish papers on relevant and clinically important topics is a paper by Odogwu *et al*, entitled *Laparoscopic cholecystectomy performed by a surgical care practitioner: a review of outcomes*, published online in April 2024 and fully in this issue.^5^ I suspect most readers will be aware of this study and the subsequent discussions it triggered, including criticism of whether the paper should have been published at all.

The full editorial independence of a scientific journal is the foundation on which its reputation is based. Clearly, the role of the *Annals* is not to set policies or regulate practice but rather to publish clinical research across the whole breadth of surgical practice and stimulate academic debate. The discussions triggered by this publication, in addition to the subsequent statements published by various national bodies, indicate the relevance and need for a wider discussion on this topic. Without this paper, the practices concerned might not have been publicised or challenged.

As editor-in-chief, I promote reader letters to afford improved clarity and understanding of published work to the wider readership. To that end, four letters were received, three of which with specific questions for Odogwu *et al*, who have kindly replied with detailed answers that we publish alongside the original paper in this issue.^6^ I would like to thank the authors of both these letters and the original paper for providing an insightful discourse from which I hope all readers will benefit.

Finally, an opening gambit in chess involves a player making a sacrifice, typically a pawn, for the sake of a compensating benefit.^7^ Similarly, the initial passionate and specific criticisms of a study should be viewed in time against the broader and longer-term merits of its publication – the ‘publishing gambit’.
